# Social and Regional Inequalities in Maternal Respiratory Syncytial Virus Vaccination in France

**DOI:** 10.1001/jamanetworkopen.2025.33530

**Published:** 2025-09-23

**Authors:** Amélie Gabet, Marion Bertrand, Marie-Joëlle Jabagi, Valérie Olié, Mahmoud Zureik

**Affiliations:** 1 Epidemiology of Health Products, French National Agency for Medicines and Health Products Safety, French National Health Insurance, Saint-Denis, France; 2 Anti-Infective Evasion and Pharmaco-Epidemiology, Center for Research in Epidemiology and Population Health, University of Paris-Saclay, Université de Versailles Saint-Quentin-en-Yvelines, University Paris-Sud, Inserm, Montigny le Bretonneux, France

## Abstract

This cross-sectional study measures the uptake of respiratory syncytial virus (RSV) among pregnant women in France in 2023 to 2024 and assesses characteristics associated with RSV vaccination.

## Introduction

Respiratory syncytial virus (RSV) is a leading cause of bronchiolitis in children younger than 2 years.^1^ A maternal bivalent recombinant vaccine containing an antigen of the RSV prefusion F protein (RSVpreF) was introduced in France in 2024 for pregnant women. Vaccination is recommended between 32 and 36 weeks’ gestation to confer passive protection to infants. France’s first vaccination campaign ran from September 15, 2024, to January 31, 2025, with full reimbursement by national health insurance. Given reported social disparities with nirsevimab in 2023 to 2024,^2^ this study aims to assess the national RSVpreF vaccination rate and compare sociodemographic and medical characteristics of vaccinated and unvaccinated women.

## Methods

This cross-sectional study followed the STROBE reporting guideline. According to French regulatory provisions (Public Health Code Articles L.1461-3 and R.1461-11), this study did not require ethics committee review or informed consent. Using the French National Health Data System,^3^ all pregnant women eligible for RSVpreF vaccination in France between September 15, 2024, and January 31, 2025, were included (eMethods in Supplement 1). Epidemiology of Health Products (EPI-PHARE) has permanent regulatory access to the National Health Data System. Women who gave birth or died before 24 weeks’ gestation, or had not reached 36 weeks’ gestation by the end of the campaign, were excluded. Continuous insurance coverage was required to limit misclassification from migration. We calculated national- and age-standardized regional vaccination rates. Vaccinated and unvaccinated women were compared on individual and geographical socioeconomic indicators using complementary solidarity health insurance (proxy of low income), French Deprivation Index (municipality level), and localized potential accessibility of general practitioners. Associations of characteristics with vaccination status were analyzed using logistic regression models including age, socioeconomic indicators, health care accessibility, region, and comorbidities to obtain odds ratios (ORs) with 95% CIs. Analysis analyses were performed using SAS Enterprise Guide software version 8.5 (SAS Institute), and a 2-sided *P* < .05 was considered significant.

## Results

Among the 264 471 pregnant women eligible for RSVpreF vaccination, 72 026 (27.2%) were vaccinated. The mean (SD) age was 30.8 (5.4) years, and 57 785 (21.9%) had complementary universal health coverage, a proxy for low income. Vaccination rates were higher among women in the least deprived municipalities (18 096 of 53 866 women [33.6%] in quintile 1) compared with the most deprived (10 691 of 48 626 women [22.0%] in quintile 5), reflecting an 11.6 percentage point deprivation gap (ie, approximately 30 700 cases). Higher uptake was observed among women aged 30 to 39 years (adjusted OR [aOR], 1.16; 95% CI, 1.13-1.18), those in socially advantaged municipalities (aOR, 1.75; 95% CI, 1.69-1.8), and those with greater health care accessibility (aOR, 1.26; 95% CI, 1.23-1.29). Lower uptake was associated with complementary solidarity health insurance status (aOR, 0.50; 95% CI, 0.49-0.51) (Table 1). Comorbidity prevalence was similar between groups. Age-standardized vaccination rates varied substantially across regions. Among 72 026 RSV-vaccinated women, 69 142 (96.1%) also received a pertussis vaccine during pregnancy, vs 126 091 of 192 445 unvaccinated women (65.5%) (Table 2).

**Table 1.** Association of Participant Characteristics With Bivalent Recombinant Respiratory Syncytial Virus Vaccination in France

Characteristic	Women, No. (%) (N = 264 471)		Vaccination rate, No./total No. (%)	Vaccination, OR (95% CI)	
				Crude	Adjusted^a^
	Vaccinated (n = 72 026)	Not vaccinated (n = 192 445)			
Age group, y					
13-17	191 (0.3)	1316 (0.7)	191/1507 (12.7)	0.49 (0.42-0.57)	0.77 (0.65-0.90)
18-24	7909 (11.0)	29 999 (15.6)	7909/37 908 (20.9)	0.77 (0.75-0.80)	0.88 (0.85-0.91)
25-29	19 821 (27.5)	56 161 (29.2)	19 821/75 982 (26.1)	1 [Reference]	1 [Reference]
30-39	40 956 (56.9)	96 364 (50.1)	40 956/137 320 (29.8)	1.20 (1.18-1.23)	1.16 (1.13-1.18)
40-50	3149 (4.4)	8605 (4.5)	3149/11 754 (26.8)	1.06 (1.01-1.11)	1.08 (1.03-1.13)
Socioeconomic indicators					
Complementary solidarity health insurance	9108 (12.6)	48 677 (25.3)	9108/57 785 (15.8)	0.46 (0.45-0.47)	0.51 (0.50-0.53)
French Deprivation Index, quintile					
First (less deprived)	18 096 (25.1)	35 770 (18.6)	18 096/53 866 (33.6)	1.79 (1.75-1.85)	1.82 (1.76-1.88)
Second	15 808 (22.0)	36 213 (18.8)	15 808/52 021 (30.4)	1.55 (1.51-1.59)	1.43 (1.39-1.47)
Third	13 703 (19.0)	35 716 (18.6)	13 703/49 419 (27.7)	1.36 (1.32-1.40)	1.29 (1.25-1.33)
Fourth	12 425 (17.3)	35 093 (18.2)	12 425/47 518 (26.1)	1.26 (1.22-1.29)	1.15 (1.12-1.19)
Fifth (more deprived)	10 691 (14.8)	37 935 (19.7)	10 691/48 626 (22.0)	1 [Reference]	1 [Reference]
Oversea territories	771 (1.1)	10 437 (5.4)	771/11 208 (6.9)	1.14 (0.93-1.39)	1.31 (1.07-1.60)
Missing	532 (0.7)	1281 (0.7)	532/1813 (29.3)	NA	NA
Health care accessibility level^b^					
1 (Lower accessibility)	21 332 (29.6)	65 848 (34.2)	21 332/87 180 (24.5)	1 [Reference]	1 [Reference]
2	23 674 (32.9)	62 702 (32.6)	23 674/83 376 (27.4)	1.15 (1.13-1.18)	1.16 (1.13-1.18)
3 (Higher accessibility)	25 784 (35.8)	59 728 (31.0)	25 784/86 512 (30.2)	1.37 (1.34-1.40)	1.26 (1.23-1.29)
Missing	1236 (1.7)	4167 (2.2)	1236/5403 (22.9)	NA	NA
Region of residence					
Auvergne-Rhône-Alpes	9358 (13.0)	22 135 (11.5)	9358/31 493 (29.7)	0.56 (0.54-0.59)	0.54 (0.52-0.57)
Bourgogne-Franche-Comté	1967 (2.7)	7069 (3.7)	1967/9036 (21.8)	0.37 (0.35-0.39)	0.41 (0.38-0.44)
Bretagne	4921 (6.8)	6563 (3.4)	4921/11 484 (42.9)	1 [Reference]	1 [Reference]
Centre-Val de Loire	2597 (3.6)	6841 (3.6)	2597/9538 (27.5)	0.51 (0.48-0.54)	0.56 (0.53-0.60)
Corse	134 (0.2)	682 (0.4)	134/816 (16.4)	0.26 (0.22-0.32)	0.24 (0.20-0.29)
Grand Est	4545 (6.3)	14 325 (7.5)	4545/18 870 (24.1)	0.42 (0.40-0.44)	0.47 (0.44-0.49)
Hauts-de-France	7335 (10.2)	17 199 (9.0)	7335/24 534 (29.9)	0.57 (0.54-0.60)	0.70 (0.67-0.73)
Ile-de-France	14 309 (19.9)	45 939 (23.9)	14 309/60 248 (23.8)	0.42 (0.40-0.43)	0.36 (0.35-0.38)
Normandie	3636 (5.1)	8571 (4.5)	3636/12 207 (29.8)	0.57 (0.54-0.60)	0.65 (0.62-0.69)
Nouvelle-Aquitaine	6902 (9.6)	12 565 (6.5)	6902/19 467 (35.5)	0.73 (0.70-0.77)	0.75 (0.72-0.79)
Occitanie	6025 (8.4)	14 805 (7.7)	6025/20 830 (28.9)	0.54 (0.52-0.57)	0.58 (0.55-0.60)
Pays de la Loire	5129 (7.1)	9317 (4.9)	5129/14 446 (35.5)	0.73 (0.70-0.77)	0.75 (0.71-0.79)
Provence-Alpes-Côte d’Azur	4487 (6.2)	15 970 (8.3)	4487/20 457 (21.9)	0.37 (0.36-0.39)	0.38 (0.36-0.40)
Comorbidities					
Obesity^c^	2959 (4.1)	9919 (5.2)	2959/12 878 (23.0)	0.82 (0.79-0.86)	0.91 (0.87-0.95)
Alcohol consumption^c^	346 (0.5)	1137 (0.6)	346/15 615 (23.3)	0.81 (0.72-0.92)	0.89 (0.78-1.01)
Tobacco smoking^c^	5654 (7.8)	15 269 (7.9)	5654/20 923 (27.0)	0.96 (0.93-1.00)	0.99 (0.95-1.02)
Opiate intake^c^	100 (0.1)	368 (0.2)	100/468 (21.4)	0.70 (0.56-0.87)	0.91 (0.73-1.15)
Cardiovascular diseases	500 (0.7)	1369 (0.7)	500/1869 (26.8)	0.96 (0.87-1.07)	0.97 (0.87-1.07)
Antihypertensive medications	913 (1.3)	2215 (1.2)	913/3128 (29.2)	1.13 (1.04-1.22)	1.12 (1.03-1.21)
Lipid-lowering medications	116 (0.2)	310 (0.2)	116/426 (27.2)	1.00 (0.80-1.24)	1.03 (0.82-1.29)
Diabetes	707 (1.0)	2039 (1.1)	707/2746 (25.7)	0.96 (0.88-1.05)	1.06 (0.97-1.16)
Cancer	605 (0.8)	1276 (0.7)	605/1881 (32.2)	1.26 (1.14-1.39)	1.14 (1.03-1.26)
Chronic respiratory diseases	2222 (3.1)	5619 (2.9)	2222/7841 (28.4)	1.08 (1.03-1.14)	1.10 (1.04-1.16)
Autoimmune diseases^d^	794 (1.1)	1980 (1.0)	794/2774 (28.6)	1.04 (0.96-1.13)	0.99 (0.91-1.08)
HIV or AIDS	111 (0.2)	329 (0.2)	111/440 (25.2)	0.93 (0.87-1.00)	1.07 (0.86-1.34)
Psychiatric illness	958 (1.3)	2737 (1.4)	958/3695 (25.9)	0.93 (0.87-0.99)	1.06 (0.92-1.22)
Psychotropic medications	3300 (4.6)	7224 (3.8)	3300/10 524 (31.4)	1.19 (1.15-1.25)	1.18 (1.13-1.24)
Neurodegenerative diseases	484 (0.7)	1378 (0.7)	484/1862 (25.9)	0.93 (0.87-1.00)	0.92 (0.82-1.03)

Abbreviation: NA, not applicable.

^a^ Adjusted for age, socioeconomic indicators, health care accessibility, region, and comorbidities.

^b^ General Practitioners’ Localized Potential Accessibility.

^c^ Any disorder related to the comorbidity.

^d^ Including inflammatory bowel diseases, rheumatoid arthritis, ankylosing spondylitis, and other chronic inflammatory diseases.

**Table 2.** Other Vaccinations Administered to Women Reaching 36 Weeks’ Gestational Age Between September 15, 2024, and January 31, 2025, According to Their Vaccination Status With the RSVpreF Vaccine

Vaccine	Women by vaccination status, No. (%)		
	Vaccinated with RSVpreF vaccine (n =72 026)	Unvaccinated with RSVpreF vaccine (n = 192 445)	Total (N = 264 471)
Pertussis vaccine	69 142 (96.1)	126 091 (65.5)	195 233 (73.8)
Seasonal influenza vaccine	26 780 (37.2)	10 432 (5.4)	37 212 (14.1)
COVID-19 vaccine	8002 (11.1)	2812 (1.5)	10 814 (4.1)
Concomitant vaccinations			
None of the listed vaccinations	2177 (3.0)	65 565 (34.1)	68 276 (25.6)
At least 1 of the listed vaccinations	69 849 (97.0)	126 880 (65.9)	196 729 (74.4)
At least 2 of the listed vaccinations	27 128 (37.7)	10 761 (5.6)	37 889 (14.3)
All 3 of the listed vaccinations,	6961 (9.7)	1680 (0.9)	8641 (3.3)

Abbreviation: RSVpreF, respiratory syncytial virus prefusion F protein.

## Discussion

This cross-sectional study found that during its first campaign in France, the national vaccination rate with RSVpreF vaccine was 27.2%, which was lower than reported rates in the US, although US data are not fully representative.^4^ Despite full reimbursement, pronounced social and geographic disparities were evident, reflecting persistent inequities in access to maternal immunization.^5^ This nationwide population-based design is well suited to studying vaccination inequities, and the national coverage of the database strengthens its relevance, despite potential misclassification in administrative data. A limitation was the unavailability of data on parity, education level, and ethnicity, which may be possible confounders of vaccine uptake. The use of municipality-level deprivation data may have limited the precision offered by finer geographic scales. Findings were consistent across models, with no collinearity and likely minimal impact from interaction effects. These disparities underscore missed opportunities for disadvantaged groups, underlying the need for targeted communication and outreach during the upcoming 2025 to 2026 vaccination campaign.

## Notes

Supplement 1.
eMethods.

Supplement 2.
Data Sharing Statement
