# Supplementary material for: Social and Regional Inequalities in Maternal Respiratory Syncytial Virus Vaccination in France
Source: JAMA Netw Open. 2025 Sep 23;8(9):e2533530. doi: 10.1001/jamanetworkopen.2025.33530 (PMC12457969; doi:10.1001/jamanetworkopen.2025.33530)
Supplement: Supplement 1. — eMethods. [file jamanetwopen-e2533530-s001.pdf]

Supplemental Online Content

Gabet A, Bertrand M, Jabagi MJ, Olié V, Zuriek M. Social and regional inequalities in maternal respiratory syncytial virus. *JAMA Netw. Open*. 2025;8(9):e2533530. doi:10.1001/jamanetworkopen.2025.33530

eMethods.

This supplemental material has been provided by the authors to give readers additional information about their work.

© 2025 Gabet A et al. *JAMA Network Open*.

eMethods

Abbreviations

ANSM, French National Agency for the Safety of Medicines and Health Products

ATC, Anatomical Therapeutic Chemical Classification System

EPI-PHARE, Scientific Interest Group in Epidemiology of Health Products

CNAM, French National Health Insurance

CNIL, National Commission on Informatics and Liberty

FDep, French Deprivation Index

PMSI, Medicalization of Information Systems Program

RSV, Respiratory Syncytial Virus

SNDS, National Health Data System

eMethods

Data source

The National Health Data System (SNDS) is a collection of pseudonymized databases providing information on the entire French population. It is primarily based on the National Information System of Inter-regime Health Insurance (SNIIRAM), which includes both data on mandatory health insurance reimbursements and data from healthcare establishments collected through the Program for the Medicalization of Information Systems (PMSI). The SNDS is further enriched by databases on mandatory maternity declaration, which provides presumed dates of pregnancy onset and dates of delivery. Data from this database have been updated monthly since January 1, 2013. As a result, we have access to all pregnancies declared in France up to January 31, 2025, as well as individual data on pregnant women from the SNDS.

This study was conducted in accordance with Articles L.1461-3 and R. 1461-11 and following the French Public Health Code, amended by Decree No. 2021-848 of June 29, 2021, related to the processing of personal data known as the "National Health Data System" (SNDS). EPI-PHARE accesses SNDS data

© 2025 Gabet A et al. *JAMA Network Open*.

through the permanent regulatory access of its supervisory agencies, the ANSM and the CNAM, in accordance with the provisions of Decree No. 2016-1871 of December 26, 2016, related to the processing of personal data known as the "National Health Data System," Articles R.1461-13 and R.1461-14 of the Public Health Code, and CNIL deliberation No. 2016-316 of the National Commission on Informatics and Liberty (CNIL). In accordance with the permanent regulatory access granted to EPI-PHARE via the ANSM and the CNAM, this work did not require specific authorization from the CNIL. This study was previously declared on the EPI-PHARE register of studies requiring the use of SNDS under reference T-2025-03-557. All queries were performed by individuals duly authorized with profile 30 or 108.

Study population

This study is a cross-sectional observational study conducted on March 1, 2025. We selected all pregnant women aged 13 to 50 between September 15, 2024 and January 31, 2025, the date on which the vaccination campaign ended. At the same time, all deliveries of RSV-vaccine Abrysvo® were searched in the DCIR databases. Women who had not theoretically reached 36 gestational age by January 31, 2025 were excluded. We also excluded women who had given birth or died before 24 of gestational age. The date of vaccination was defined as the date of the pharmacist's vaccination act, a medical consultation, or a midwife's or nurse's act performed within 30 days after vaccine delivery. If none were found, the dispensing date was used. Women were classified as vaccinated before 32 weeks, between 32–36 weeks (recommended window), or after 36 weeks of gestation.

Collected Data

For each woman, we collected individual characteristics (age, health insurance scheme, region of residence), pregnancy details (pregnancy start date, delivery date if applicable), vaccination status, comorbidities, and socio-economic indicators such as complementary solidarity health insurance, the 2020 version of the French deprivation index Fdep (1), and Localized Potential Accessibility of the General Practitioner (2). For vaccinated women, gestational age at vaccination and the vaccine

© 2025 Gabet A et al. *JAMA Network Open*.

prescriber's specialty (general practitioner, gynecologist/obstetrician, midwife, or other) were recorded. We also identified deliveries of other recommended vaccines during pregnancy and available in France over the 2024-2025 season: influenza (Fluarix Tetra®, Vaxigrip Tetra®, Influvac Tetra®, Efluelda Tetra® (available until 04/04/2024 only)) and pertussis vaccines (Repevax®, Boostrix tetra®) via their billing codes, and the COVID-19 vaccine available in the 2024–2025 season (Comirnaty JN.1® 30 µg), based on specific billing codes (e.g., COVID-19 vaccination remuneration or injection acts).

Analyses

As a sensitivity analysis, we stratified the logistic regression models by health care accessibility level. The associations between socioeconomic indicators and vaccination remained consistent across strata. All analyses were performed using the SAS Entreprise Guide software, version 8.5.

References

1. Rey G, Jougla E, Fouillet A, Hémon D. Ecological association between a deprivation index and mortality in France over the period 1997 - 2001: variations with spatial scale, degree of urbanicity, age, gender and cause of death. BMC Public Health. 2009;9:33.
2. Barlet M, Coldefy M, Collin C, Lucas-Gabrielli V. Local Potential Accessibility (LPA): A New Measure of Accessibility to Private General Practitioners. Health economics Journal (The Research, Studies, Evaluation and Statistics Department). 2012. Number 174. Available at: https://www.irdes.fr/EspaceAnglais/Publications/IrdesPublications/QES174.pdf.

© 2025 Gabet A et al. *JAMA Network Open*.
