# Supplementary material for: Social and Regional Inequalities in Maternal Respiratory Syncytial Virus Vaccination in France
Source: JAMA Netw Open. 2025 Sep 23;8(9):e2533530. doi: 10.1001/jamanetworkopen.2025.33530 (PMC12457969; doi:10.1001/jamanetworkopen.2025.33530)
Supplement: Supplement 2. — Data Sharing Statement [file jamanetwopen-e2533530-s002.pdf]

Data Sharing Statement

Gabet. Social and Regional Inequalities in Maternal Respiratory Syncytial Virus Vaccination in France. *JAMA Netw Open*. Published September 23, 2025.
doi:10.1001/jamanetworkopen.2025.33530

Data
Data available: No

Additional Information
Explanation for why data not available: Data are not available. According to data protection and the French regulation, the authors cannot publicly release the data from the French national health data system (SNDS). However, any person or organization, public or private, for-profit or non-profit, is able to access SNDS data upon authorization from the French Data Protection Office (CNIL) to carry out a study, a research, or an evaluation of public interest.
